# Supplementary figures and images for: Hepatic AMPK signaling dynamic activation in response to REDOX balance are sentinel biomarkers of exercise and antioxidant intervention to improve blood glucose control
Source: eLife. 2022 Sep 26;11:e79939. doi: 10.7554/eLife.79939 (PMC9645808; doi:10.7554/eLife.79939)

Figure 2\_source data\_01

2B

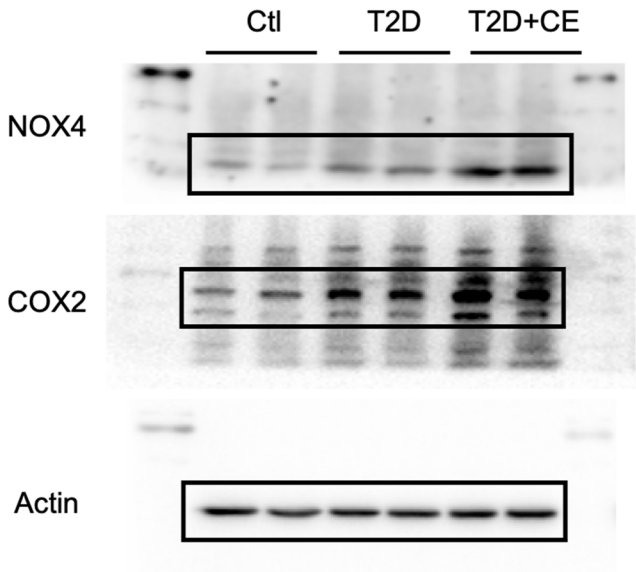

2E

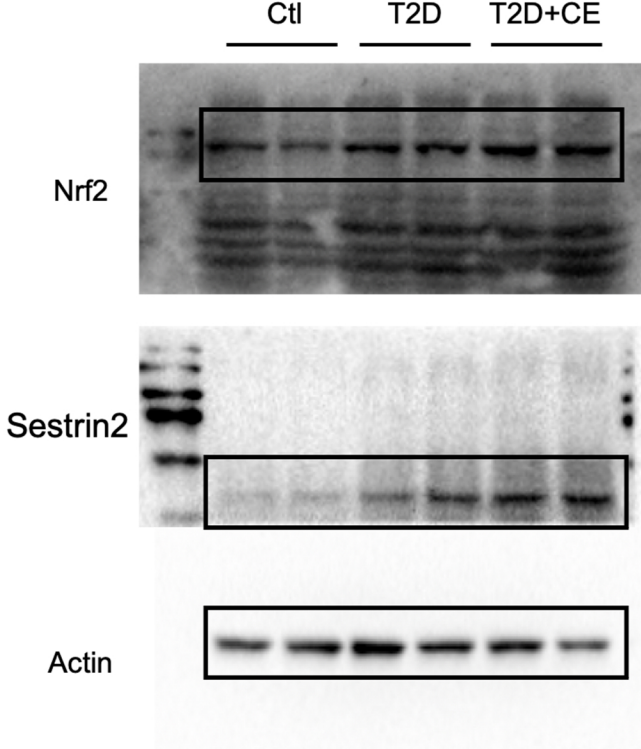

2H

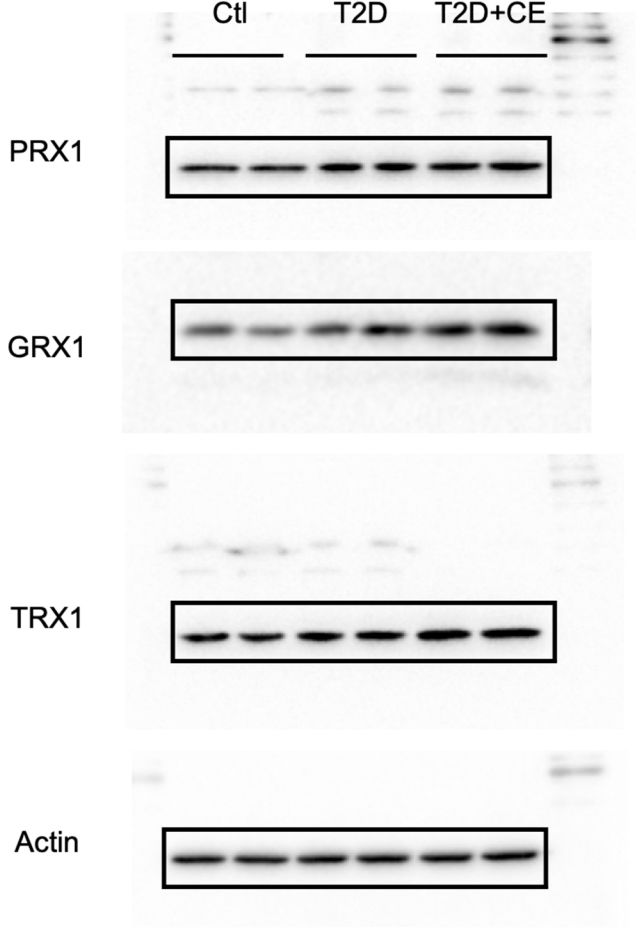

2M

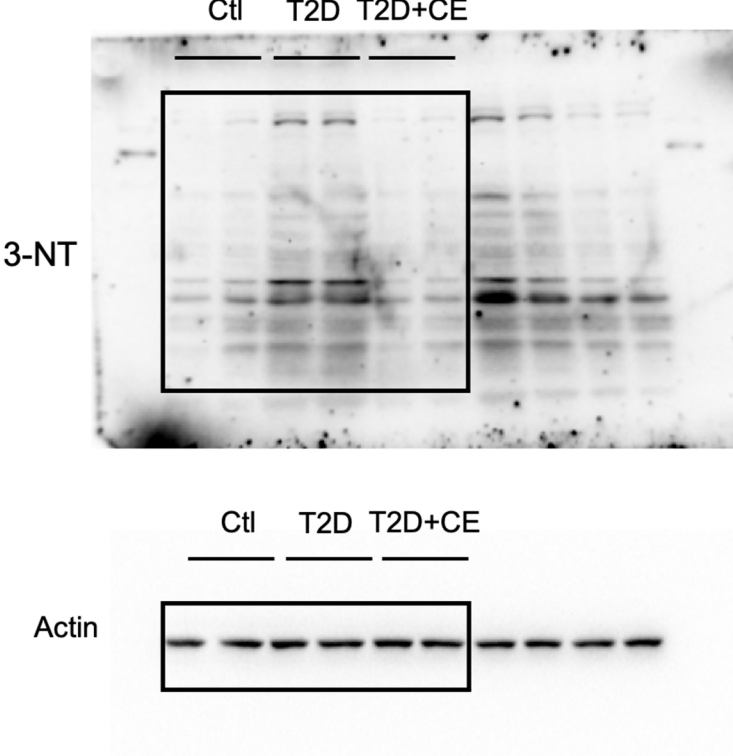

Supplement: Figure 2—source data 1. [file elife-79939-fig2-data1.pdf]

Figure 3\_source data\_01

3E

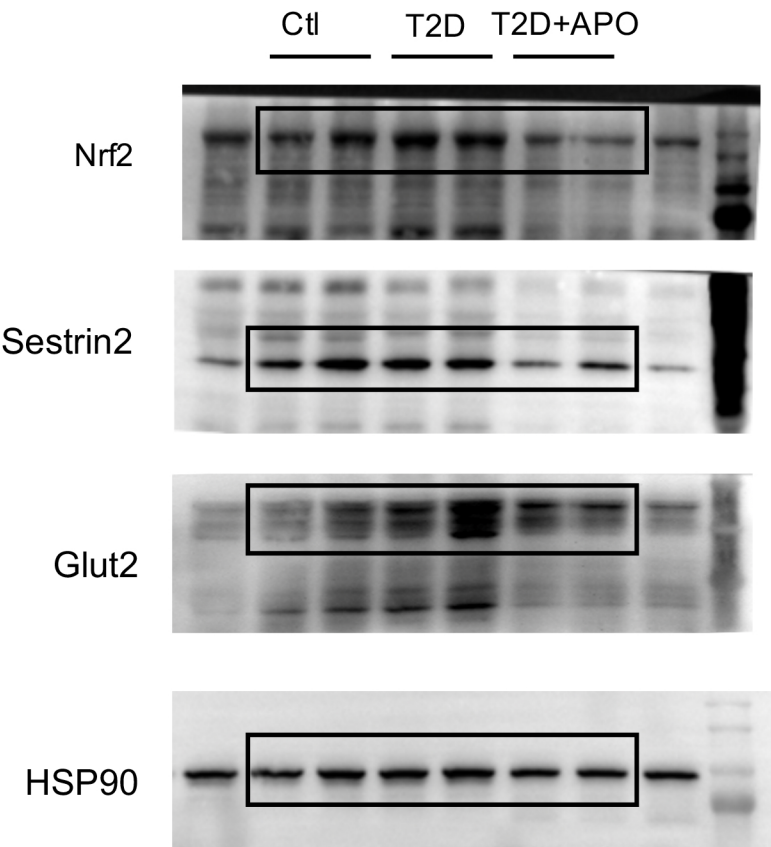

Supplement: Figure 3—source data 1. [file elife-79939-fig3-data1.pdf]

Figure 4\_source data\_01

4C

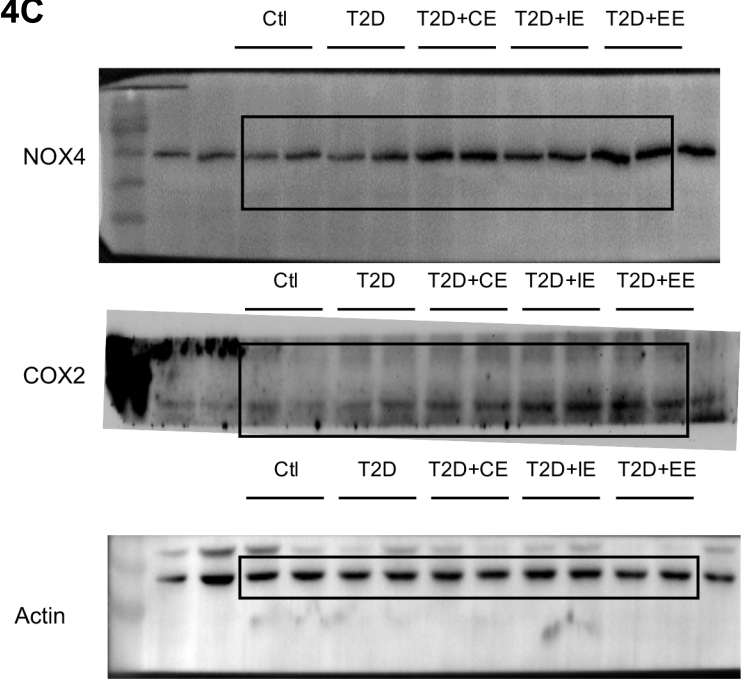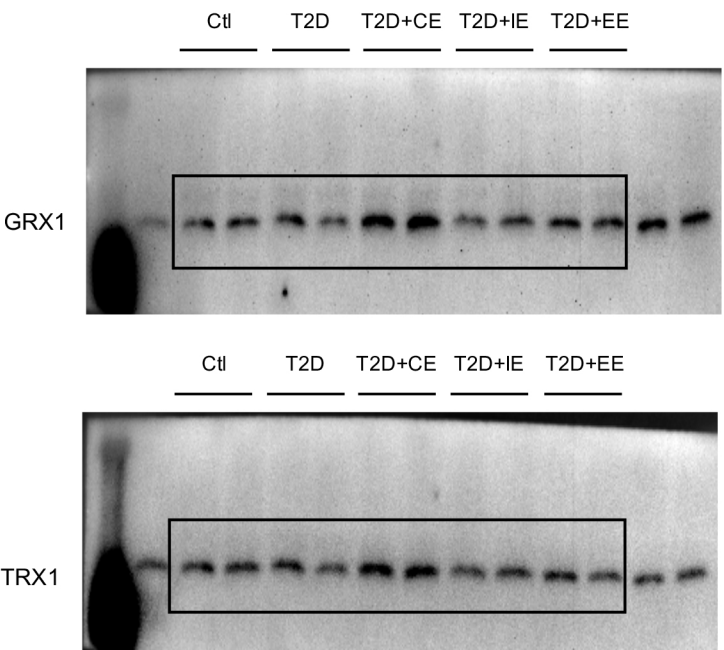

4F

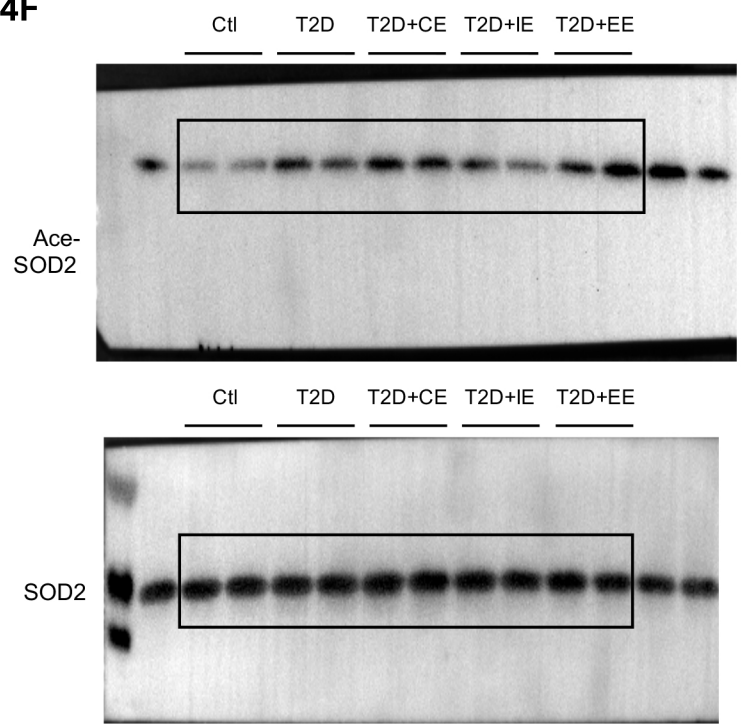

4O

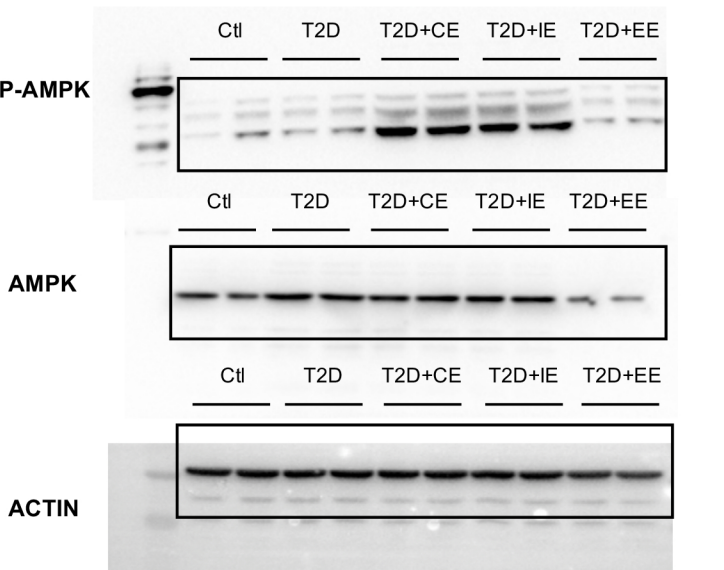

Supplement: Figure 4—source data 1. [file elife-79939-fig4-data1.pdf]

Figure 5\_source data\_01

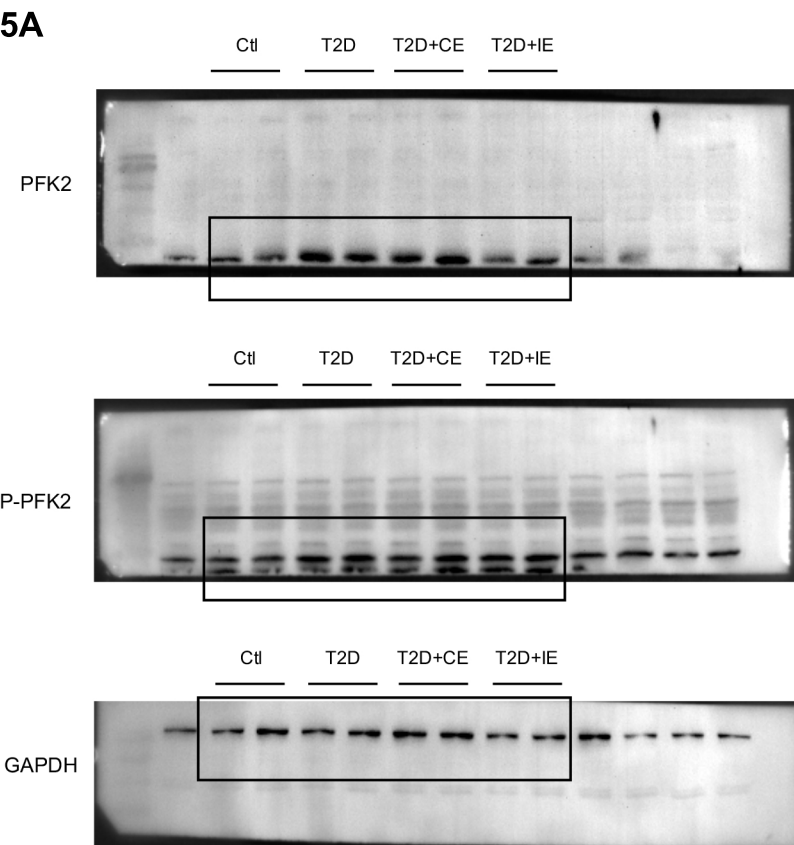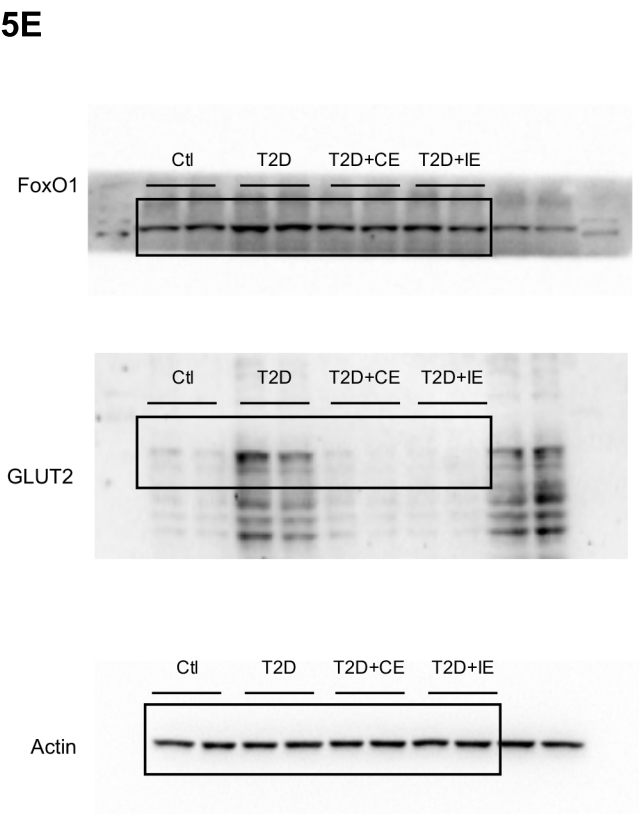

Supplement: Figure 5—source data 1. [file elife-79939-fig5-data1.pdf]

Figure 6\_source data\_01

6A

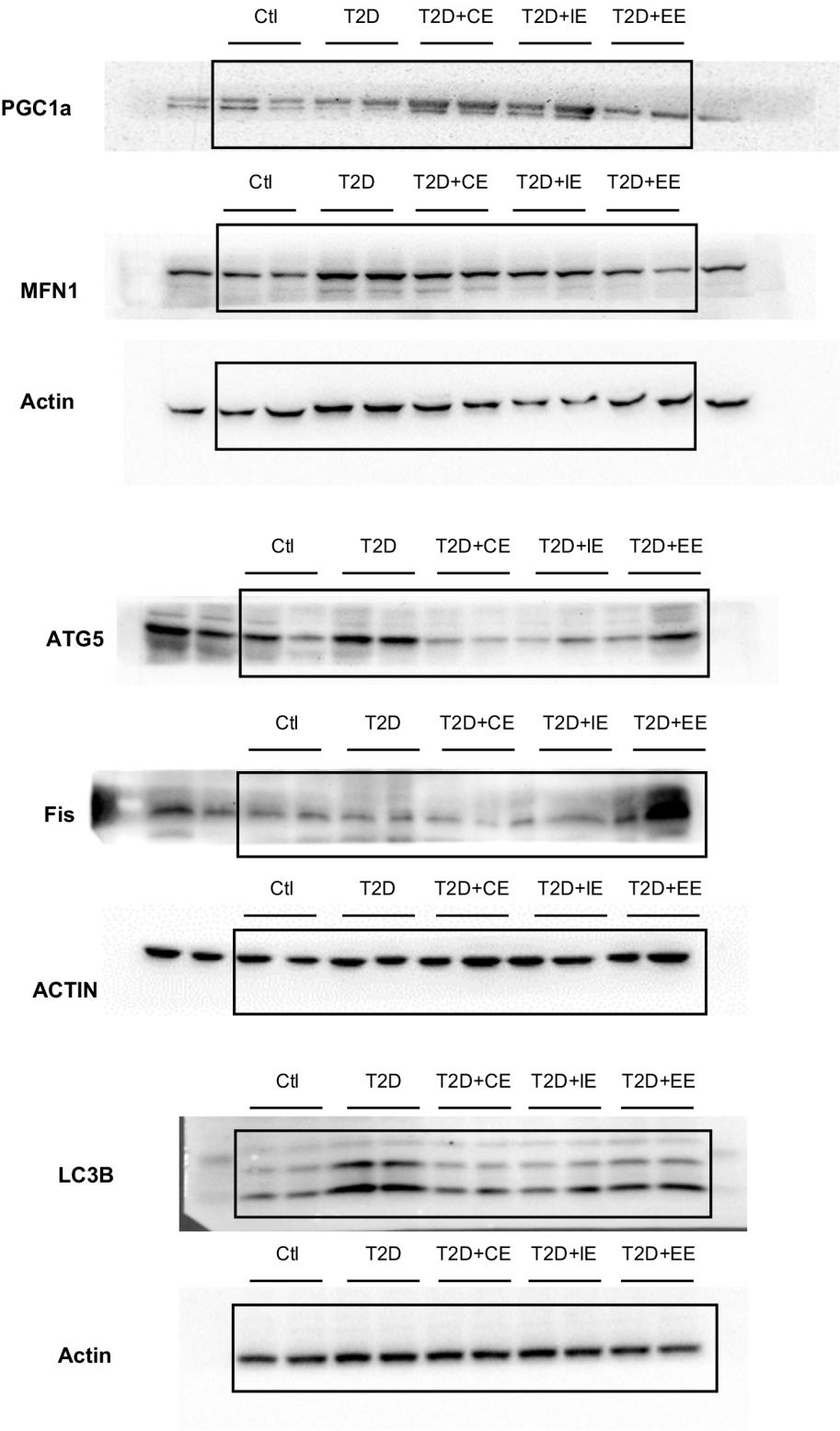

Supplement: Figure 6—source data 1. [file elife-79939-fig6-data1.pdf]

7D

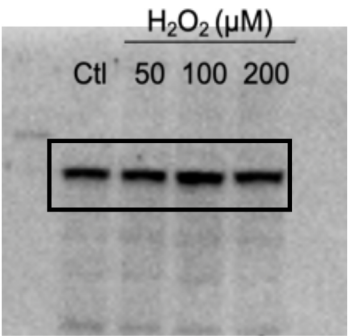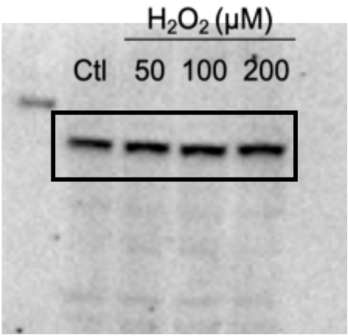

7E

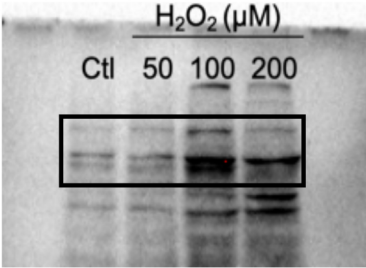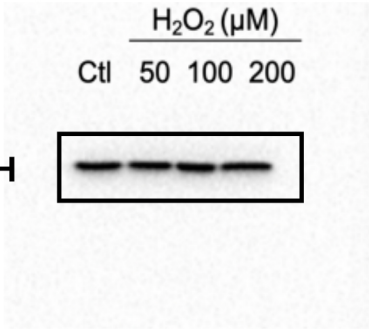

7C

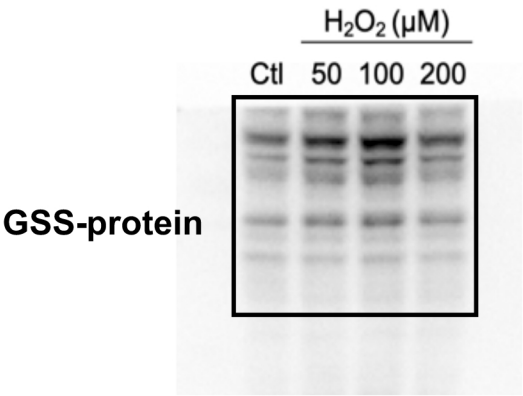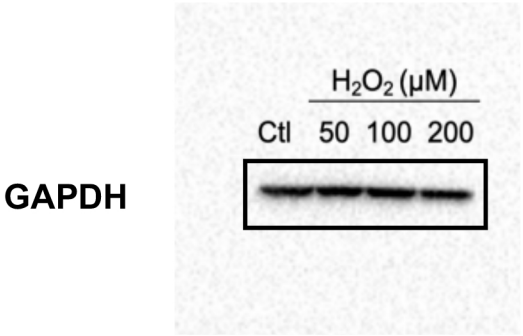

Supplement: Figure 7—source data 1. [file elife-79939-fig7-data1.pdf]
