# Supplementary material for: Hepatic AMPK signaling dynamic activation in response to REDOX balance are sentinel biomarkers of exercise and antioxidant intervention to improve blood glucose control
Source: eLife. 2022 Sep 26;11:e79939. doi: 10.7554/eLife.79939 (PMC9645808; doi:10.7554/eLife.79939)
Supplement: Supplementary file 1. [file elife-79939-supp1.docx]

**Supplementary file 1a** Antibody information

|  | Name | Supplier | Cat No. |
| --- | --- | --- | --- |
| 1 | CAT | ProteinTech (Wuhan, CN) | 21260-1-AP |
| 2 | PRX1 | ProteinTech (Wuhan, CN) | 15816-1-AP |
| 3 | AMPKa1 | ProteinTech (Wuhan, CN) | 10929-2-AP |
| 4 | GRX1 | ProteinTech (Wuhan, CN) | 15804-1-AP |
| 5 | GRX2 | ProteinTech (Wuhan, CN) | 13381-1-AP |
| 6 | SOD2 | ProteinTech (Wuhan, CN) | 24127-1-AP |
| 7 | HSP90 | ProteinTech (Wuhan, CN) | 13171-1-AP |
| 8 | COX1 | ProteinTech (Wuhan, CN) | 13393-1-AP |
| 9 | COX2 | ProteinTech (Wuhan, CN) | 12375-1-AP |
| 10 | PFK2 | ProteinTech (Wuhan, CN) | 17838-1-AP |
| 11 | P-AMPKa1/a2 | SAB | #11183 |
| 12 | 3-NT | Abcam | ab61392 |
| 13 | 4HNE | Abcam | ab46545 |
| 14 | NOX4 | Abcam | Ab133303 |
| 15 | PGC1-α | Abcam | ab54481 |
| 16 | Actin | Cell Signaling Technology (USA) | #3700 |
| 17 | Acetylated-Lysine | Cell Signaling Technology (USA) | #9441 |
| 18 | P-PFK2 | Cell Signaling Technology (USA) | #13064 |
| 19 | Ace-SOD2 | ProteinTech (Wuhan, CN) |  |
| 20 | ATG5 | Cell Signaling Technology (USA) | #12994 |
| 21 | LC3A/B | Cell Signaling Technology (USA) | #12741 |
| 22 | Anti-rabbit IgG-HRP | Cell Signaling Technology (USA) | #7074 |
| 23 | Anti-mouse IgG-HRP | Cell Signaling Technology (USA) | #7076 |
| 24 | GAPDH | Cell Signaling Technology (USA) | #2118 |
| 25 | MFN1 | Cell Signaling Technology (USA) | #14739 |
| 26 | Fis1 | Merck | ABC67 |

**Supplementary file 1b** Chow and HFD diet nutrition composition

|  | **Chow** | **HFD** |
| --- | --- | --- |
| **Composition** | **(%)** | **(%)** |
| Water (%) | 9.2 | 8.6 |
| Crude protein (%) | 22.1 | 18.8 |
| Crude fat (%) | 5.28 | 16.2 |
| Crude ash (%) | 5.2 | 5.2 |
| Corase fiber (%) | 4.12 | 3.98 |
| Nitrogen free extract (%) | 52 | 45.2 |
| Calcium (%) | 1.24 | 1.24 |
| Phosphorus (%) | 0.92 | 0.83 |
| Calcium : Phosphorus | 1.35 | 1.49 |
| Lysine (%) | 1.34 | 1.38 |
| Methionine + dicysteine (%) | 0.72 | 0.78 |
| Calorie (kcal) | 352 | 379 |
